# Synthesis and Fungicidal Activity of Novel 2,3-Disubstituted-1,3-benzoxazines

**DOI:** 10.3390/molecules17078174

**Published:** 2012-07-06

**Authors:** Zilong Tang, Zhonghua Zhu, Zanwen Xia, Hanwen Liu, Jinwen Chen, Wenjing Xiao, Xiaoming Ou

**Affiliations:** 1 School of Chemistry and Chemical Engineering, Hunan University of Science and Technology, Xiangtan 411201, China; Email: hua206@163.com (Z.Z.); xiazanwen163@163.com (Z.X.); hwyliu@126.com (H.L.); lyr1976@163.com (J.C.); wenjing_xiao@yahoo.com (W.X.); 2 Key Laboratory of Theoretical Chemistry and Molecular Simulation of Ministry of Education, Hunan University of Science and Technology, Xiangtan 411201, China; Email: xmouhn@163.com; 3 Jiangxi Dongbang Pharmaceutical Co. Ltd., Fengxin 330700, China; 4 National Engineering Research Center for Agrochemicals, Hunan Research Institute of Chemical Industry, Changsha 410014, China

**Keywords:** 2,3-disubstituted-1,3-benzoxazine, synthesis, chlorotrimethylsilane, fungicidal activity

## Abstract

A series of new 2,3-disubstituted-3,4-dihydro-2*H*-1,3-benzoxazines were prepared in moderate to excellent yields by aza-acetalizations of aromatic aldehydes with 2-(*N*-substituted aminomethyl)phenols in the presence of TMSCl. Their structures were confirmed by IR, ^1^H-NMR, ^13^C-NMR, MS and elemental analysis. The fungicidal activities of the target compounds were preliminarily evaluated, and some compounds exhibited good activity against *Rhizoctonia solani*.

## 1. Introduction

3,4-dihydro-2*H*-1,3-benzoxazines exhibit a wide range of biological activity [1,2,3,4,5,6,7,8,9,10,11], such as bactericidal, fungicidal, antitumour, antituberculosis, and anthelmintic effects, therefore, the synthesis of these compounds has attracted great interest. Several elegant methods for the preparation of these compounds have been documented in the literature [12,13,14,15,16,17,18,19,20]. Burke and co-workers disclosed a Mannich-type condensation of phenols with primary amines and formaldehyde to provide 2-unsubstituted 3,4-dihydro-2*H*-1,3-benzoxazines [5,12,13,14]. Condensations of 2-aminomethylphenol with aliphatic aldehydes or ketones provided another route to 3,4-dihydro-2*H*-1,3-benzoxazines [15,16,17]. It was noted that condensation reactions could be operated without catalyst, but sometimes a catalyst such as TsOH or triethylamine was necessary. Recently, rhodium-catalyzed reactions of 2-(alkenyloxy)benzylamines which involve an allylic cleavage followed by regioselective carbonylation at the internal carbon atom have been developed as a new way to generate 3,4-dihydro-1,3-benzoxazines [19,20]. Despite these advances, the synthesis of novel 3,4-dihydro-2*H*-1,3-benzoxazines and the search for more efficient routes for drug discovery and medicinal chemistry are still highly desirable. In our previous paper [21], a new method by SnCl_4_-mediated aza-acetalization reactions of aromatic aldehydes with 2-arylaminomethyl phenols to synthesize substituted 3,4-dihydro-2*H*-1,3-benzoxazines was developed and the compounds showed good fungicidal activity. Herein, we present the synthesis of a series of novel 2-aryl-3-alkyl-3,4-dihydro-2*H*-1,3-benzoxazines, as a continuation of our ongoing project aimed at searching for novel biological active nitrogen and oxygen linked heterocyclic compounds, by reactions of aromatic aldehydes with 2-(*N*-substituted aminomethyl)-phenols in the presence of chlorotrimethylsilane (TMSCl) [22,23,24,25], and also report their fungicidal activities.

## 2. Results and Discussion

### 2.1. Chemistry

**Scheme 1:**
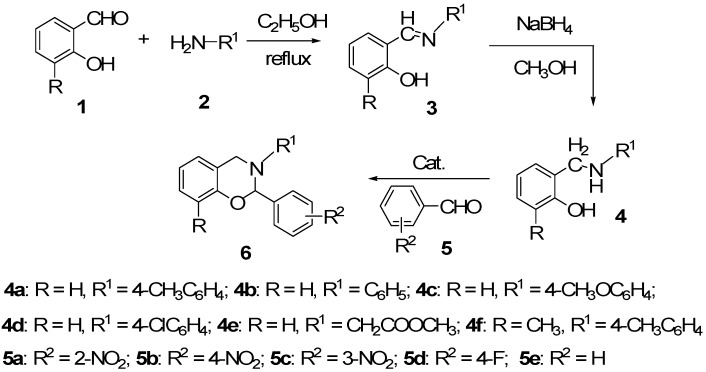
Synthesis of 2,3-disubstituted 3,4-dihydro-2*H*-1,3-benzoxazines **6**.

The synthetic route to the title compounds **6a–n** is shown in Scheme 1. Initially, the reaction of fluorobenzaldehyde (**5d**) with 2-((4-methylphenyl)aminomethyl)phenol (**4a**) which was prepared in high yield by reaction of salicylaldehyde and *p*-toluidine followed by reduction with NaBH_4_ in a one-pot process [21,26,27] was chosen as model reaction for the synthesis of the title compounds **6a–n**. The reaction was carried out in a mixed solvent of chloroform and cyclohexane (v:v = 1:2) under reflux in the presence of TMSCl (20 mol%) by removing the water of condensation azeotropically, and the desired product **6a** was obtained in 57% yield (Table 1, entry 1). It should be noted that the interest in preparation of fluorine-containing 3,4-dihydro-2*H*-1,3-benzoxazines is due to the special structure and biological character of fluorine atom, which was usually introduced in drugs and pesticides to enhance or change the biological activity.

Then, under the same conditions, compounds **6b–n** were further prepared by reactions of aromatic aldehydes **5a–e** with 2-(*N*-substituted aminomethyl)phenols **4a–f**, and all the experimental results are listed in Table 1. The results clearly showed that all reactions gave the desired products in moderate to excellent yields. It was observed that the reactions of nitrobenzaldehydes furnished the products in higher yields than those with fluorobenzaldehyde or benzaldehyde.

Moreover, the reactions of *N*-alkyl substituted aminomethylphenols gave higher yields than those of *N*-aryl substituted ones. The lower yield of the latter can be attributed to its low nucleophilicity, which was in turn caused by the conjugation effect between the electron pair on the nitrogen atom and the aryl group. All these results indicated apparently that TMSCl was an efficient catalyst for the reactions, and to the best of our knowledge, this is the first time to adopt TMSCl as catalyst for aza-acetalizations of aromatic aldehydes with 2-aminomethylphenols to synthesize 3,4-dihydro-2*H*-1,3-benzoxazines.

**Table 1:** The results of the preparation of 1,3-benzoxazines 6 ^a^.

Entry	R	R^1^	R^2^	Product	Yield/% ^b^
1	H	4-CH_3_C_6_H_4_	4-F	**6a**	57
2	H	C_6_H_5_	4-F	**6b**	55
3	H	4-CH_3_OC_6_H_4_	4-F	**6c**	53
4	H	4-ClC_6_H_4_	4-F	**6d**	59
5	H	CH_2_COOCH_3_	4-F	**6e**	62
6	H	4-ClC_6_H_4_	3-NO_2_	**6f**	67
7	H	4-ClC_6_H_4_	H	**6g**	57
8	CH_3_	4-CH_3_C_6_H_4_	2-NO_2_	**6h**	75
9	CH_3_	4-CH_3_C_6_H_4_	3-NO_2_	**6i**	78
10	CH_3_	4-CH_3_C_6_H_4_	4-NO_2_	**6j**	78
11	H	C_6_H_5_	4-NO_2_	**6k**	73
12	H	CH_2_COOCH_3_	2-NO_2_	**6l**	88
14	H	CH_2_COOCH_3_	3-NO_2_	**6n**	90

^a^ The mole ratio of n (aromatic aldehyde **5**)/n (*o*-aminomethyl phenol **4**) = 1.3:1 for all reactions. TMSCl: 20 mol% based on aminomethyl phenol. CHCl_3_/C_6_H_12_ = 1:2 (v:v). Reaction time: 5 h. Temperature: 85 °C. ^b^ Isolated yield.

The structures of the products were established on the basis of their spectroscopic data (IR, ^1^H-NMR, ^13^C-NMR, MS) and elemental analysis [21]. All compounds exhibit characteristic signals appropriately (see experimental section). This can be illustrated with compound **6l**. In the IR spectrum, a strong absorption at 1731 cm^−1^ corresponds to the stretching vibration of the C=O group, 1524 and 1365 cm^−1^ relate to the NO_2_ group, and 1607, 1585 cm^−1^ to the C=C bond. A singlet at 6.57 observed in the ^1^H-NMR spectrum corresponds to the OCHN proton of the benzoxazine ring. The downfield shift of this OCHN proton is due to the strong electronegativity of the nitrogen and oxygen atoms. Particularly, the NCH_2_ proton absorbs as two doublets at 3.78 and 4.14 instead of a singlet. Meanwhile, the mass spectrum (ESI-MS) displays a molecular ion peak at *m/z* 346 [M+NH_4_]^+^.

### 2.2. Fungicidal Activity Assay

According to standard operation procedure (SOP) developed by Hunan Branch of National Pesticide R&D South Center of China [28], fungicidal activities of the prepared compounds **6a–n** against *Gibberella zeae*, *Phytophythora capsici*, *Alternaria alternate*, *Botrytis cinerea* and *Sclerotonia sclerotiorum* were evaluated using the mycelium growth rate test in concentration of 25 µg/mL, which was expressed as inhibition rate (%), and their activities against *Rhizoctonia solani* using the leaf-disc culture in concentration of 500 µg/mL, which was expressed as control efficacy (%). The results are summarized in Table 2. In general, the results demonstrated that most of the compounds displayed moderate to good activity. Compounds **6k**, **6l**, **6n** showed 100% activity against *Rhizoctonia solani*. But, compared with compounds**6l** (R^1^ = CH_2_COOCH_3_, R^2^ = 2-NO_2_) and **6n** (R^1^ = CH_2_COOCH_3_, R^2^ = 3-NO_2_), the activity of the isomer **6m** (R^1^ = CH_2_COOCH_3_, R^2^ = 4-NO_2_) dramatically decreased to 0%. Similarly, the activity against *Rhizoctonia solani* and *Sclerotonia sclerotiorum* of compound **6h** with a methyl group on the position-6 of benzoxazine ring (R = Me) dramatically decreased to 0% relative to the compound **6o** (R = H, 100%, 60%) [21]. Also, the activity against *Rhizoctonia solani* and *Phytophythora capsici* of compound **6i** (R = Me) decreased to 0% and 3% compared with **6p** (R = H, 50%, 37%). But, the activity against *Sclerotonia sclerotiorum* of compound 6i increased to 52% compared with 6p (0%). In addition, some compounds displayed good activity against *Sclerotonia sclerotiorum* as shown by **6k** (91%), **6d** (89%), **6f** (89%), **6n** (83%) and **6a** (81%).

**Table 2:** Fungicidal activity of compounds **6a–n**.

Compd.	*Phytophythor a capsici* /%	*Gibberella zeae* /%	*Sclerotonia sclerotiorum* /%	*Alternaria alternata* /%		*Botrytis cinerea* /%		*Rhizoctonia solani* /%	
**6a**	39	40	81	13		28		0	
**6b**	21	37	69	0		29		0	
**6c**	18	40	28	0		14		0	
**6d**	55	49	89	21		52		0	
**6e**	21	35	31	0		7		0	
**6f**	24	40	89	25		46		0	
**6g**	27	40	48	17		51		0	
**6h**	9	26	0	8		0		0	
**6i**	3	23	52	13		7		0	
**6j**	0	16	37	21		14		50	
**6k**	0	0	91	0		25		100	
**6l**	0	0	52	0		12		100	
**6m**	0	33	37	13		19		0	
**6n**	0	0	83	25		19		100	
**6o ^a^**	28	31	60	11		19		100	
**6p ^a^**	37	10	0	18		14		50	

^a^ The preparation of **6o** (R = H, R^1^ = 4-CH_3_C_6_H_4_, R^2^ = 2-NO_2_) and 6p (R = H, R^1^ = 4-CH_3_C_6_H_4_, R^2^ = 3-NO_2_) see reference [21].

## 3. Experimental

### 3.1. Materials and Reagents

All solvents were dried by standard procedure. Aromatic aldehydes and substituted anilines were commercially available. Infrared spectra were recorded on a PE-2000 FT-IR. ^1^H- and ^13^C-NMR spectra were recorded on a Bruker Avance-500 MHz spectrometer. Chemical shifts (δ) are given relative to Me_4_Si (0, ^1^H) or CDCl_3_ (77.0, ^13^C). Mass spectra were obtained with Thermo Finnigan LCQ Advantage spectrometer. Elemental analysis was measured on PE 2400 II CHNS instrument. Melting points were determined on a WRS-1B digital melting point instrument. Thin-layer chromatography (TLC) was run on precoated silica gel phates (Merck 60F_254_).

### 3.2. Chemical Synthesis

#### 3.2.1. Synthesis of 2-(*N*-Substituted aminomethyl) Phenols **4a–f** [21,26,27]

*2-((4-Methylphenylamino)methyl)phenol* (**4a**): Yield 91%. White solid, m.p.: 120.5–121.2 °C; ^1^H-NMR (CDCl_3_) *δ*: 2.30 (s, 3H, CH_3_), 4.42 (s, 2H, CH_2_), 6.79 (d, 2H, *J* = 8.5 Hz), 6.88-6.93 (m, 2H), 7.08 (d, 2H, *J* = 8.0 Hz), 7.16 (d, 1H, *J* = 7.5 Hz), 7.23 (t, 1H, *J* = 7.45 Hz); ^13^C-NMR (CDCl_3_) *δ*: 20.60, 49.34, 116.25 (2C), 116.67, 119.98, 122.98, 128.67, 129.19, 129.91 (2C), 130.46, 144.64, 157.00; IR (KBr, cm^−1^) ν: 3435, 3260, 3032, 3011, 2977, 2861, 2734, 1614, 1592, 1512, 1467, 1456, 1402, 1291, 1249, 1232, 1187, 1110, 1057, 976, 911, 863, 834, 820, 801, 788, 753, 742, 719, 706.

*2-((Phenylamino)methyl)phenol* (**4b**): Yield 85%. White solid, m.p.: 129.4–130.8 °C; ^1^H-NMR (CDCl_3_) *δ*: 4.45 (s, 2H, CH_2_), 6.87–6.97 (m, 5H), 7.18 (d, 1H, *J* = 7.5 Hz), 7.24~7.30 (m, 3H); ^13^C-NMR (CDCl_3_) *δ*: 48.71, 115.93 (2C), 116.66, 120.13, 120.85, 122.99, 128.78, 129.26, 129.43 (2C), 147.22, 156.76; IR (KBr, cm^−1^) ν: 3445, 3264, 30652, 2854, 1594, 1499, 1459, 1436, 1389, 1358, 1316, 1301, 1266, 1251, 1237, 1184, 1166, 1114, 1088, 1056, 1040, 1025, 971, 903, 841, 796, 754, 727, 689.

*2-((4-Methoxyphenylamino)methyl)phenol* (**4c**): Yield 85%. Purple solid, m.p.: 132.1–133.8 °C; ^1^H-NMR (CDCl_3_) *δ*: 3.78 (s, 3H, OCH_3_), 4.40 (s, 2H, CH_2_), 6.83–6.87 (m, 4H), 6.88~6.93 (m, 2H), 7.14 (d, 1H, *J* = 7 Hz), 7.23 (t, 1H, *J* = 7.5 Hz); ^13^C-NMR (CDCl_3_) *δ*: 50.24, 55.66, 114.77 (2C), 116.67, 117.85 (2C), 119.87, 122.78, 128.58, 129.18, 140.39, 154.61, 157.17; IR (KBr, cm^−1^) ν: 3444, 3253, 3000, 2956, 2862, 1714, 1637, 1593, 1510, 1468, 1457, 1409, 1358, 1289, 1249, 1225, 1177, 1112, 1058, 1033, 979, 909, 864, 830, 788, 759, 742, 717.

*2-((4-Chlorophenylamino)methyl)phenol* (**4d**): Yield 89%. White solid, m.p.: 121.7–122.4 °C; ^1^H-NMR (CDCl_3_) *δ*: 4.40 (s, 2H, CH_2_), 6.77 (d, *J* = 9Hz, 2H), 6.90 (t, *J* = 6.5 Hz, 2H), 7.17–7.28 (m, 4H); ^13^C-NMR (CDCl_3_) *δ*: 48.42, 116.62, 116.89 (2C), 120.31, 122.66, 125.52, 128.86, 129.28 (2C), 129.38, 145.77, 156.39; IR (KBr, cm^−1^) ν: 3435, 3257, 3013, 2969, 2938, 2729, 2626, 1594, 1492, 1462, 1454, 1403, 1392, 1357, 1314, 1285, 1250, 1232, 1181, 1120, 1109, 1097, 1060, 1008, 974, 907, 866, 844, 829, 815, 796, 770, 758, 667.

*2-((3-Methoxycarbonylmethylamino)methyl)phenol* (**4e**): Yield 74%. White solid, m.p.: 84.9–85.9 °C; ^1^H-NMR (CDCl_3_) *δ*: 3.46 (s, 2H), 3.76 (s, 3H), 4.00 (s, 2H), 6.77~6.80 (m, 1H), 6.85 (d, *J* = 8.0 Hz, 1H), 6.9 8(d, *J* = 7.0 Hz, 1H), 7.17 (t, *J* = 7.5 Hz, 1H); ^13^C-NMR (CDCl_3_) *δ*: 48.57, 51.99, 52.07, 116.44, 119.19, 121.72, 128.66, 129.00, 157.81, 171.83; IR (KBr, cm^−1^) ν: 3451, 3352, 2894, 2857, 2118, 1898, 1735, 1616, 1587, 1484, 1429, 1369, 1302, 1260, 1224, 1206, 1185, 1136, 1104, 1037, 988, 929, 899, 866, 847, 756, 720.

*2-((4-Methylphenylamino)methyl)-6-methylphenol* (**4f**): Yield: 85%. White solid, m.p.: 81.0–81.7 °C; ^1^H-NMR (CDCl_3_) *δ*: 2.24 (s, 3H), 2.28 (s, 3H), 4.37 (s, 2H), 6.77 (t, *J* = 7.5Hz, 3H), 6.98 (d, *J* = 7.5 Hz, 1H), 7.05 (dd, *J* = 8.0 Hz, *J* = 7.5 Hz, 3H), 8.93 (s, 1H, OH); ^13^C-NMR (CDCl_3_) *δ*: 15.73, 20.51, 49.34, 116.14 (2C), 119.32, 122.08, 125.47, 126.13, 129.77 (2C), 130.29, 130.33, 144.54, 155.07; IR (KBr, cm^−1^) ν : 3421, 3335, 2919, 2853, 2731, 1714, 1615, 1592, 1517, 1471, 1446, 1432, 1314, 1259, 1237, 1217, 1123, 1085, 1051, 1012, 930, 883, 822, 812, 762.

#### 3.2.2. Synthesis of 3,4-Dihydro-2*H*-1,3-benzoxazines **6a–n**

*General Procedure*: Under nitrogen, into a 250 mL three-necked flask equipped with a Dean-Stark trap, 2-(benzaminomethyl)phenol (**4b**, 0.99 g, 5 mmol), 4-nitrobenzaldehyde (**5b**, 0.98 g, 6.5 mmol), a mixed solvent of chloroform and cyclohexane (150 mL, v:v = 1:2), and TMSCl (0.11 g, 20 mol%) were added with stirring. The solution was heated at 85 °C for 5 h (checked by TLC), and the water of condensation was removed by azeotropic distillation of most of solvent. Then, triethylamine was added to make solution pH = 8, followed by addition of ethyl acetate (100 mL), and the mixture was washed sequentially with water (2 × 100 mL) and saturated brine (2 × 100 mL). The organic phase was dried over Na_2_SO_4_, and evaporated under reduced pressure. The obtained yellow oil was purified by recrystallization from ethyl acetate-petroleum ether giving the product **6k** (73% yield) as a yellow solid.

*2-(4-Fluorophenyl)-3-p-tolyl-3,4-dihydro-2H-1,3-benzoxazine* (**6a**): Yield: 57%. White solid, m.p.: 66.5–66.9 °C; ^1^H-NMR (CDCl_3_) *δ*: 2.27 (s, 3H, CH_3_), 4.29 (s, 2H), 6.54 (s, 1H), 6.82–6.88 (m, 2H), 6.95 (d, *J* = 8.5 Hz, 1H), 7.00 (t, *J* = 8.5 Hz, 2H), 7.07 (s, 4H), 7.13 (t, *J* = 7.0 Hz, 1H), 7.50 (t, *J* = 6.0 Hz, 2H); ^13^C-NMR (CDCl_3_) *δ*: 20.67, 46.59, 88.17, 115.39, 115.56, 116.90, 120.47, 120.60 (2C), 120.70, 126.61, 128.08, 128.55, 128.61, 129.82, 131.95, 135.02 (d, *J*_CF_ = 3.0 Hz), 147.30, 152.83, 161.55, 163.51; IR (KBr, cm^−1^) ν: 3427, 2922, 2869, 2339, 1612, 1585, 1514, 1505, 1456, 1382, 1339, 1232, 1217, 1194, 1154, 1128, 1034, 975, 949, 898, 819, 753, 714; MS (ESI): 320 [M+H]^+^. Anal. Calcd for C_21_H_18_FNO: C, 78.98; H, 5.68; N, 4.39; Found: C, 78.46; H, 5.64; N, 4.42.

*2-(4-Fluorophenyl)-3-phenyl-3,4-dihydro-2H-1,3-benzoxazine* (**6b**): Yield: 55%. White solid, m.p.: 85.0–86.2 °C; ^1^H-NMR (CDCl_3_) *δ*: 4.33 (d, *J* = 4.5 Hz, 2H), 6.61 (s, 1H), 6.83–6.89 (m, 2H), 6.97–7.04 (m, 4H), 7.14–7.19 (m, 3H), 7.26–7.29 (m, 2H), 7.50–7.53 (m, 2H); ^13^C-NMR (CDCl_3_) *δ*: 46.14, 87.59, 115.37, 115.54, 116.86, 120.09, 120.29, 120.68, 122.18, 126.51, 128.06, 128.43, 128.49, 129.24, 134.78 (d, *J*_CF_ = 3.0 Hz), 149.58, 152.61, 156.67, 161.47, 163.43; IR (KBr, cm^−1^) ν: 3040, 2959, 2853, 2369, 1942, 1899, 1601, 1581, 1509, 1495, 1451, 1394, 1346, 1293, 1226, 1158, 1125, 1110, 1033, 1014, 976, 952, 937, 822, 764, 697; Anal. Calcd for C_20_H_16_FNO: C, 78.67; H, 5.28; N, 4.59; Found: C, 78.24; H, 5.31; N, 4.56.

*2-(4-Fluorophenyl)-3-(4-methoxyphenyl)-3,4-dihydro-2H-1,3-benzoxazine* (**6c**): Yield: 53%. White solid, m.p.: 76.9–77.4 °C; ^1^H-NMR (CDCl_3_) *δ*: 3.74 (s, 3H, OCH_3_), 4.27 (d, *J* = 4.0 Hz, 2H), 6.42 (s, 1H), 6.78 (d, *J* = 9.0 Hz, 2H), 6.85–6.88 (m, 2H), 6.96–7.03 (m, 3H), 7.10–7.16 (m, 3H), 7.51–7.54 (m, 2H); ^13^C-NMR (CDCl_3_) *δ*: 47.37, 55.40, 88.83, 114.25, 114.63, 115.25, 115.42, 116.78, 117.93, 120.66, 122.92, 126.54, 127.98, 128.52, 128.58, 129.16, 134.86 (d, *J*_CF_ = 3.1 Hz), 143.08, 152.89, 161.43, 163.39; IR (KBr, cm^−1^) ν: 3256, 2954, 2911, 1839, 2052, 1908, 1870, 1605, 1581, 1509, 1490, 1456, 1437, 1379, 1346, 1240, 1230, 1153, 1105, 1038, 1019, 980, 956, 894, 836, 759, 605; Anal. Calcd for C_21_H_18_FNO_2_: C, 75.21; H, 5.41; N, 4.18; Found: C, 75.53; H, 5.39; N, 4.20.

*2-(4-Fluorophenyl)-3-(4-chlorophenyl)-3,4-dihydro-2H-1,3-benzoxazine* (**6d**): Yield: 59%. White solid, m.p.: 80.7–81.3 °C; ^1^H-NMR (CDCl_3_) *δ*: 4.29 (s, 2H), 6.51 (s, 1H), 6.83-6.87 (m, 2H), 6.95 (d, *J* = 8.0 Hz, 1H), 6.99 (t, *J* = 8.5 Hz, 2H), 7.08 (d, *J* = 8.5 Hz, 2H), 7.14 (t, *J* = 7.0 Hz, 1H), 7.19 (d, *J* = 8.5 Hz, 2H), 7.46–7.49 (m, 2H); ^13^C-NMR (CDCl_3_) *δ*: 46.65, 87.58, 115.44, 115.61, 116.89, 119.89, 120.88, 121.68, 126.53, 127.33, 128.24, 128.38, 128.44, 129.15 (2C), 134.35 (d, *J*_CF_ = 3.1 Hz), 148.09, 152.47, 161.52, 163.48; IR (KBr, cm^−1^) ν: 3436, 3059, 2955, 1894, 1710, 1605, 1584, 1507, 1488, 1457, 1381, 1342, 1224, 1158, 1022, 1006, 982, 959, 952, 838, 830, 763, 724; Anal. Calcd for C_20_H_15_ClFNO: C, 70.69; H, 4.45; N, 4.12; Found: C, 70.37; H, 4.47; N, 4.09.

*Methyl 2-(2-(4-Fluorophenyl)-2H-1,3-benzoxazin-3(4H)-yl)acetate* (**6e**): Yield: 62%. White solid, m.p.: 119.8–120.3 °C; ^1^H-NMR (CDCl_3_) *δ*: 3.42 (s, 2H), 3.68 (s, 3H, CH_3_), 3.94 (d, *J* = 17.0 Hz, 1H), 4.25 (d, *J* = 17.0 Hz, 1H), 5.95 (s, 1H), 6.89–6.98 (m, 3H), 7.05–7.08 (m, 2H), 7.16–7.20 (m, 1H), 7.59–7.62 (m, 2H); ^13^C-NMR (CDCl_3_) *δ*: 49.47, 49.91, 51.84, 89.87, 115.23, 115.42, 116.63, 119.07, 121.06, 127.66, 128.02, 128.59, 128.66, 133.46 (d, *J*_CF_ = 3.0 Hz), 133.48, 153.30, 171.36; IR (KBr, cm^−1^) ν: 3472, 3084, 3061, 2956, 2909, 1909, 1747, 1607, 1582, 1510, 1487, 1450, 1389, 1341, 1310, 1248, 1219, 1157, 1138, 1107, 1032, 1000, 992, 948, 903, 861, 827, 761; MS (ESI): 319 [M+NH_4_]^+^. Anal. Calcd for C_17_H_16_FNO_3_: C, 67.76; H, 5.35; N, 4.65; Found: C, 67.42; H, 5.32; N, 4.63.

*2-(3-Nitrophenyl)-3-(4-chlorophenyl)-3,4-dihydro-2H-1,3-benzoxazine* (**6f**): Yield: 67%. Yellow solid, m.p.: 145.1–145.8 °C; ^1^H-NMR (CDCl_3_) *δ*: 4.26 (d, *J* = 17.0 Hz, 1H), 4.36 (d, *J* = 17.0 Hz, 1H), 6.55 (s, 1H), 6.87 (d, *J* = 4.5 Hz, 2H), 7.02 (d, *J* = 8.0 Hz, 1H), 7.13 (d, *J* = 8.5 Hz, 2H), 7.17 (q, *J* = 4.5 Hz, 1H), 7.21 (d, *J* = 8.5 Hz, 2H), 7.52 (t, *J* = 8.0 Hz, 1H), 7.88 (d, *J* = 8.0 Hz, 1H), 8.16 (d, *J* = 8.5 Hz, 1H), 8.43 (s, 1H); ^13^C-NMR (CDCl_3_) *δ*: 47.25, 87.02, 117.08, 119.54, 121.34, 122.11 (3C), 123.34, 126.59, 127.98, 128.53, 129.26 (2C), 129.76, 132.91, 140.99, 147.78, 148.59, 152.00; IR (KBr, cm^−1^) ν: 3444, 3074, 3040, 2973, 2873, 1884, 1732, 1594, 1583, 1521, 1495, 1455, 1386, 1348, 1231, 1198, 1131, 1095, 1034, 990, 954, 893, 824, 808, 757, 725, 706; Anal. Calcd for C_20_H_15_ClN_2_O_3_: C, 66.49; H, 4.12; N, 7.64; Found: C, 66.68; H, 4.14; N, 7.61.

*3-(4-Chlorophenyl)-2-phenyl-3,4-dihydro-2H-1,3-benzoxazine* (**6g**): Yield: 57%. White solid, m.p.: 108.6–108.8 °C; ^1^H-NMR (CDCl_3_) *δ*: 4.27 (d, *J* = 16.5 Hz, 1H), 4.32 (d, *J* = 16.5 Hz, 1H), 6.57 (s, 1H), 6.83-6.88 (m, 2H), 6.98 (d, *J* = 8.0 Hz, 1H), 7.11 (d, *J* = 7.0 Hz, 2H), 7.14 (t, *J* = 8.5 Hz, 1H), 7.20 (d, *J* = 9.0 Hz, 2H), 7.28–7.36 (m, 3H), 7.51 (d, *J* = 8.0 Hz, 2H); ^13^C-NMR (CDCl_3_) *δ*: 46.55, 88.02, 116.58, 116.87, 120.06, 120.19, 120.71, 121.48, 126.52, 127.10, 128.16, 128.59, 128.96, 129.12, 129.33, 129.72, 134.44, 138.69, 148.29, 152.75; IR (KBr, cm^−1^) ν: 3432, 3044, 2980, 1887, 1711, 1609, 1575, 1500, 1479, 1368, 1346, 1220, 1141, 1036, 1001, 968, 854, 836, 831, 768, 720; Anal. Calcd for C_20_H_16_ClNO: C, 74.65; H, 5.01; N, 4.35; Found: C, 75.98; H, 4.98; N, 4.33.

*8-Methyl-2-(2-nitrophenyl)-3-p-tolyl-3,4-dihydro-2H-1,3-benzoxazine* (**6h**): Yield: 75%. Yellow solid, m.p.: 138.3–139.3 °C; ^1^H-NMR (CDCl_3_) *δ*: 2.24 (s, 3H, CH_3_), 2.32 (s, 3H,CH_3_), 3.98 (d, *J* = 17.0 Hz, 1H), 4.19 (d, *J* = 17.0 Hz, 1H), 6.68 (d, *J* = 7.5 Hz, 1H), 6.75 (t, *J* = 7.0 Hz, 1H), 7.03 (t, *J* = 9.0 Hz, 6H), 7.43–7.46 (m, 2H), 7.49–7.51 (m, 1H), 7.72–7.73 (m, 1H); ^13^C-NMR (CDCl_3_) *δ*: 15.81, 20.65, 47.04, 85.43, 119.65, 120.29, 120.70 (2C), 124.05, 124.35, 125.60, 128.28, 129.08, 129.34, 129.64 (2C), 131.79, 132.49, 132.99, 146.69, 148.92, 150.36; IR (KBr, cm^−1^) ν: 3433, 3082, 2981, 2918, 1611, 1594, 1531, 1514, 1468, 1439, 1389, 1365, 1224, 1200, 1144, 968, 820, 766, 735; Anal. Calcd for C_22_H_20_N_2_O_3_: C, 73.32; H, 5.59; N, 7.77; Found: C, 73.59; H, 5.56; N, 7.73.

*8-Methyl-2-(3-nitrophenyl)-3-p-tolyl-3,4-dihydro-2H-1,3-benzoxazine* (**6i**): Yield: 78%. Yellow solid, m.p.: 118.4–118.7 °C; ^1^H-NMR (CDCl_3_) *δ*: 2.28 (s, 3H, CH_3_), 2.37 (s, 3H, CH_3_), 4.25 (d, *J* = 17.0 Hz, 1H), 4.35 (d, *J* = 17.0 Hz, 1H), 6.61 (s, 1H), 6.71 (d, *J* = 7.0 Hz, 1H), 6.75 (t, *J* = 7.0 Hz, 1H), 7.03 (d, *J* = 7.0 Hz, 1H), 7.07–7.12 (m, 4H), 7.51 (t, *J* = 8.0 Hz, 1H), 7.86 (d, *J* = 7.5 Hz, 1H), 8.15-8.17 (m, 1H), 8.43 (s, 1H); ^13^C-NMR (CDCl_3_) *δ*: 15.86, 20.62, 46.98, 87.55, 119.39, 120.41, 120.77 (2C), 122.01, 123.16, 124.02, 125.99, 129.31, 129.65, 129.80 (2C), 132.29, 132.75, 141.66, 146.99, 148.59, 150.19; IR (KBr, cm^−1^) ν: 3434, 3090, 3026, 2917, 2856, 1714, 1612, 1595, 1579, 1528, 1514, 1472, 1451, 1378, 1345, 1222, 1194, 1127, 1079, 998, 967, 940, 811, 767, 730, 691; Anal. Calcd for C_22_H_20_N_2_O_3_: C, 73.32; H, 5.59; N, 7.77; Found: C, 73.64; H, 5.56; N, 7.74.

*8-Methyl-2-(4-nitrophenyl)-3-p-tolyl-3,4-dihydro-2H-1,3-benzoxazine* (**6j**): Yield: 78%. Yellow solid, m.p.: 130.1–130.9 °C; ^1^H-NMR (CDCl_3_) *δ*: 2.27 (s, 3H, CH_3_), 2.35 (s, 3H, CH_3_), 4.22 (d, *J* = 17.0 Hz, 1H), 4.35 (d, *J* = 17.0 Hz, 1H), 6.62 (s, 1H), 6.71 (d, *J* = 7.0 Hz, 1H), 6.75 (t, *J* = 7.5 Hz, 1H), 7.03 (d, *J* = 7.0 Hz, 1H), 7.06-7.10 (m, 4H), 7.69 (d, *J* = 8.5 Hz, 2H), 8.19 (d, *J* = 8.5 Hz, 2H); ^13^C-NMR (CDCl_3_) *δ*: 15.84, 20.60, 47.13, 87.71, 119.40, 120.43, 120.62 (2C), 123.82, 124.06, 124.27, 125.83, 127.63, 129.29, 129.79 (2C), 130.46, 132.24, 146.51, 146.89, 147.66, 150.29; IR (KBr, cm^−1^) ν: 3436, 3024, 2963, 2919, 2855, 1608, 1596, 1517, 1469, 1384, 1347, 1227, 1198, 1128, 1083, 1013, 957, 903, 855, 845, 834, 761, 738, 721; Anal. Calcd for C_22_H_20_N_2_O_3_: C, 73.32; H, 5.59; N, 7.77; Found: C, 73.01; H, 6.02; N, 7.74.

*2-(4-Nitrophenyl)-3-phenyl-3,4-dihydro-2H-1,3-benzoxazine* (**6k**): Yield: 73%. Yellow solid, m.p.: 117.2–118.8 °C; ^1^H-NMR (CDCl_3_) *δ*: 4.25 (d, *J* = 17.0 Hz, 1H), 4.40 (d, *J* = 17.0 Hz, 1H), 6.64 (s, 1H), 6.87 (d, *J* = 7.5 Hz, 2H), 7.00–7.03 (m, 2H), 7.17–7.21 (m, 3H), 7.26–7.31 (m, 2H), 7.74 (d, *J* = 8.5 Hz, 2H), 8.19 (d, *J* = 7.0 Hz, 2H); ^13^C-NMR (CDCl_3_) *δ*: 46.72, 87.22, 116.51, 116.95, 119.96, 120.28, 121.15, 122.68, 123.84, 124.26, 126.60, 127.86, 128.31, 129.34 (2C), 130.46, 146.28, 147.68, 149.21, 152.21; IR (KBr, cm^−1^) ν: 3444, 3087, 3056, 3038, 3007, 2970, 2912, 1707, 1596, 1581, 1522, 1492, 1453, 1388, 1346, 1230, 1208, 1144, 1109, 1034, 978, 958, 888, 853, 828, 759, 741; Anal. Calcd for C_20_H_16_N_2_O_3_: C, 72.28; H, 4.85; N, 8.43; Found: C, 72.59; H, 4.83; N, 8.39.

*Methyl 2-(2-(2-nitrophenyl)-2H-1,3-benzoxazin-3(4H)-yl)acetate* (**6l**): Yield: 88%. White solid, m.p.: 108.6–109.0 °C; ^1^H-NMR (CDCl_3_) *δ*: 3.38 (s, 2H), 3.66 (s, 3H, CH_3_), 3.78 (d, *J* = 17.5 Hz, 1H), 4.14 (d, *J* = 17.0 Hz, 1H), 6.57 (s, 1H), 6.94–7.00 (m, 3H), 7.21–7.24 (m, 1H), 7.49–7.53 (m, 1H), 7.57-7.60 (m, 1H), 7.81-7.84 (m, 2H); ^13^C-NMR (CDCl_3_) *δ*: 48.99, 51.33, 51.92, 87.19, 116.58, 119.18, 121.39, 124.71, 127.86, 128.20, 128.26, 129.41, 131.95, 132.16, 148.86, 152.95, 170.57; IR (KBr, cm^−1^) ν: 3446, 3010, 2958, 2881, 1953, 1912, 1731, 1607, 1585, 1524, 1488, 1461, 1444, 1424, 1365, 1275, 1263, 1222, 1122, 1109, 1034, 1002, 963, 780, 761, 742; MS (ESI): 346 [M+NH_4_]^+^. Anal. Calcd for C_17_H_16_N_2_O_5_: C, 62.19; H, 4.91; N, 8.53; Found: C, 62.47; H, 4.88; N, 8.49.

*Methyl 2-(2-(4-nitrophenyl)-2H-1,3-benzoxazin-3(4H)-yl)acetate* (**6m**): Yield: 91%. White solid, m.p.: 137.2–138.9 °C; ^1^H-NMR (CDCl_3_) *δ*: 3.37 (s, 2H), 3.71 (s, 3H, CH_3_), 3.94 (d, *J* = 17.0 Hz, 1H), 4.21 (d, *J* = 17.0 Hz, 1H), 6.03 (s, 1H), 6.92–7.00 (m, 3H), 7.20 (t, *J* = 7.0 Hz, 1H), 7.84 (d, *J* = 8.5 Hz, 2H), 8.24 (d, *J* =8.5 Hz, 2H); ^13^C-NMR (CDCl_3_) *δ*: 49.11, 50.41, 51.97, 89.34, 116.68, 118.80, 121.45, 123.68 (2C), 127.72, 127.92 (2C), 128.26, 144.90, 147.82, 152.70, 171.04; IR (KBr, cm^−1^) ν: 3468, 3079, 3038, 2854, 1745, 1609, 1580, 1523, 1488, 1447, 1420, 1384, 1346, 1313, 1221, 1134, 1109, 992, 952, 904, 826, 764; Anal. Calcd for C_17_H_16_N_2_O_5_: C, 62.19; H, 4.91; N, 8.53; Found: C, 62.50; H, 4.89; N, 8.57.

*Methyl 2-(2-(3-nitrophenyl)-2H-1,3-benzoxazin-3(4H)-yl)acetate* (**6n**): Yield: 90%. White solid, m.p.: 161.6–162.3 °C; ^1^H-NMR (CDCl_3_) *δ*: 3.38 (s, 2H), 3.70 (s, 3H, CH_3_), 3.96 (d, *J* = 17.0 Hz, 1H), 4.23 (d, *J* = 17.0 Hz, 1H), 6.03 (s, 1H), 6.93–7.01 (m, 3H), 7.20 (t, *J* = 7.5 Hz, 1H), 7.56 (t, *J* = 7.5 Hz, 1H), 7.98 (d, *J* = 7.5 Hz, 1H), 8.19 (d, *J* = 8.0 Hz, 1H), 8.52 (s, 1H); ^13^C-NMR (CDCl_3_) *δ*: 49.28, 50.32, 51.96, 89.17, 116.80, 118.85, 121.47, 122.18, 123.44, 127.69, 128.30, 129.58, 133.06, 140.10, 148.48, 152.77, 171.02; IR (KBr, cm^−1^) ν: 3431, 2957, 2905, 1756, 1607, 1582, 1525, 1486, 1456, 1440, 1418, 1379, 1343, 1250, 1216, 1197, 1184, 1129, 1110, 1002, 956, 914, 757, 685; Anal. Calcd for C_17_H_16_N_2_O_5_: C, 62.19; H, 4.91; N, 8.53; Found: C, 62.49; H, 4.93; N, 8.56.

### 3.3. Biological Assay [28]

The *in vitro* inhibition of the title compounds against five strains of phytopathogenic fungi *Phytophythora capsici*, *Gibberella zeae*, *Sclerotonia sclerotiorum*, *Alternaria alternata and Botrytis cinerea* was performed according to standard method NY/T1156.5–2006, and antifungal activity assays adopted drug-containing medium method. Stock solution of every test compound was prepared in DMF (20 g/L) and then diluted to the required test concentrations (500 mg/L) with water containingTween 80 (0.4 mg/L). Solutions of the test compounds (2 mL) were added to potato dextrose agar(PDA) medium (38 mL, 45 °C) to provide the final concentration of 25 mg/L. The mixed mediumwithout sample was used as the blank control. The inocula, 6.5 mm in diameter, were removed fromthe margins of actively growing colonies of mycelium, placed in the centers of the above plates. Four replicates per treatment. Percentages of growth inhibition were calculated by comparing the mean value of the diameters of the mycelia in the test plates after placing in 28 °C biochemical incubator thermostat for 4 days. The inhibition percent was calculated according to the following equation:


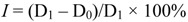


where *I* is the inhibition rate, D_1_ is the average diameter of myceliain the blank test, and D_0_ is the average diameter of mycelia in the presence of compounds. The results are given in Table 2.

*Activity against Rhizoctonia solani*. Compounds tested for control of rice sheath blight pathogen, *Rhizoctonia solani*, on rice seedlings at the fifth-leaf stage were formulated in water and DMF (5 + 1 by volume) (containing 2.5 g/L Tween 80) to 500 mg/L solutions, and applied to the rice seedlings as foliar sprays using a hand-held spray gun. The next day the seedlings were inoculated with the chaff medium within *Rhizoctonia solani* (the causal fungus of the rice sheath blight). Then the plantswere immediately placed in a temperature- and humidity-controlled chamber at 28 °C for 4 days. After treatment, percentage of disease control in the treated seedlings was compared to that of seedlings with a treatment in the absence of the experimental compounds, and fungicidal activity was estimated. Four replicates were included in the evaluation, and the biological effect was reported as the average of the four replicates. The results are given in Table 2. 

## 4. Conclusions

In summary, we have demonstrated TMSCl is an efficient catalyst for aza-acetalizations of aromatic aldehydes with 2-(*N*-substituted aminomethyl)phenols, and a series of novel 2,3-disubstituted-3,4-dihydro-2*H*-1,3-benzoxazines **6a–n** were prepared in moderate to excellent yields. The fungicidal activities of the prepared compounds were preliminarily evaluated, and some compounds exhibited good activity against *Rhizoctonia solani* as shown by **6k**, **6l**, **6n** (100% at concentration of 500 μg/mL), and some compounds displayed good activity against *Sclerotonia sclerotiorum* as shown by **6a**, **6d**, **6f**, **6k** and**6n** (81–91% at concentration of 25 μg/mL).

## References

[1] Mireya E.R., Carrajal M.A., Rincon J.M. (1980). Synthesis of some benzoxazines and the study of their possible antibacterial activity. Rev. Colomb. Cienc. Quim. Farm..

[2] Gomez P.G., Pabon H.P., Carvajal M.A., Rincon J.M. (1985). Syntesis de cuatro benzoxazinas y determinacion de su expectro de actividad antibacteriana. Rev. Colomb. Cienc. Quim. Farm..

[3] Waisser K., Gregor K., Kubicova L., Klimesova V., Kunes J., Machacek M., Kaustova J. (2000). New groups of antimycobacterial agents: 6-chloro-3- phenyl-4-thioxo-2H-1,3-benzoxazine-2(3H)-ones and 6-chloro-3-phenyl-2H-1,3-benzoxazine -2,4(3H)-dithiones. Eur. J. Med. Chem..

[4] Waisser K., Gregor K., Dostal H., Kunes J., Kubicova L., Klimesova V., Kaustova J. (2001). Influence of the replacement of the *oxo* function with the thioxo group on the antimycobacterial activity of 3-aryl-6,8-dichloro-2H-1,3-benzoxazine-2,4(3H)-diones and 3-arylquinazoline-2,4(1H,3H)-diones. Il Farmaco.

[5] Mathiew B.P., Kumar A., Sharma S., Shula P.K., Nath M. (2010). An eco-friendly synthesis and antimicrobial activities of dihydro-2H- benzo-and naphtho-1,3-oxazine derivatives. Eur. J. Med. Chem..

[6] Chylinska J.B., Urbanski T., Mordarski M. (1963). Dihydro-1,3-oxazine Derivatives and their Antitumor Activity. J. Med. Chem..

[7] Bouaziz Z., Riondel J., Mey A., Berlion M., Villard J., Filliond H. (1991). Synthesis of some naphthoxazine carbolactone derivatives with *in vitro* cytotoxic and antifungal activities synthesis of some naphthoxazine carbolactone derivatives with in vitro cytotoxic and antifungal activities. Eur. J. Med. Chem..

[8] Benameur L., Bouaziz Z., Nebois P., Bartoli M.H., Boitard M., Fillion H. (1996). Synthesis of furonaphth[1,3]oxazine and furo[1,3]oxazinoquinoline derivatives as precursors for an o-quinonemethide structure and potential antitumor agents. Chem. Pharm. Bull..

[9] Wang S., Li Y., You Q., Liu Y., Lu A. (2008). Novel hexacyclic camptothecin derivatives. Part 1: Synthesis and cytotoxicity of camptothecins with an A-ring fused 1,3-oxazine ring. Bioorg. Med. Chem. Lett..

[10] Pasternak A., Goble S.D., Struthers M., Vicario P.P., Ayala J.M., Salvo J.D., Kilburn R., Wisniewski T., DeMartino J.A., Mills S.G. (2010). Discovery of a potent and orally bioavailable CCR2 and CCR5 dual antagonist. ACS Med. Chem. Lett..

[11] Petrlikova E., Waisser K., Divišova H., Husakova P., Vrabcova P., Kuneš J., Kolar K., Stolarikova J. (2010). Highly active antimycobacterial derivatives of benzoxazine. Bioorg. Med. Chem..

[12] Burke W.J. (1949). 3,4-Dihydro-1,3,2*H*-Benzoxazines. Reaction of p-substituted phenols with *N*,*N*-dimethylol-amines. J. Am. Chem. Soc..

[13] Burke W.J., Murdock K.C., Ec G. (1954). Condensation of hydroxyaromatic compounds with formaldehyde and primary aromatic amines. J. Am. Chem. Soc..

[14] Rivera A., Ospina E., Sanchez A., Joseph-Nathan P. (1986). Synthesis of 2,2’-ethylene-bis(1,2-dihydrobenzo[h]-3H-4,2-benzoxazine) and 3,3′-ethylene(3,4- dihydrobenzo[h]-2H-1,3-benzoxazine) and assignation of their ^1^H-NMR spectra using the LAOCN3computer program. Heterocycles.

[15] McDonagh A.F., Smith H.E. (1968). Ring-chain tautomerism of derivatives of o-hydroxybenzylamine with aldehydes and ketones. J. Org. Chem..

[16] Neuvonen K., Pihlaja K. (1988). Studies on the benzoxazine series. Part 1. Preparation and ^1^H and ^13^C nuclear magnetic resonance structural study of some substituted 3,4-dihydro-2*H*-1,3-benzoxazines. J. Chem. Soc. Perkin. Trans. II.

[17] Szatmari I., Martinek T.A., Lazar L., Fulop F. (2004). Synthesis of 2,4-diaryl-3,4-dihydro-2*H*-naphth[2,1-*e*][1,3]oxazines and Study of the Effects of the Substituents on Their Ring-Chain Tautomerism. Eur. J. Org. Chem..

[18] Colin J.L., Loubinoux B. (1982). Nouvelle voie d'acces aux dihydro-3,4-2H-benzoxazines-1,3. Tetrahedron Lett..

[19] Campi E.M., Jackson W.R., McCubbin Q.J., Trnacek A.E. (1994). Allylic rearrangements during the rhodium-catalysed reactions of 2-allyloxybenzylamines and 2-(*N*-allyl-*N*-benzylamino)benzylamin. J. Chem. Soc. Chem. Commun..

[20] Campi E.M., Jackson W.R., McCubbin Q.J., Trnacek A.E. (1996). The stereochemistry of organometallic compounds. XLIII. Rhodium-catalysed reactions of 2-(alkenyloxy) benzylamines and 2-(*N*-Allyl-*N*-benzylamino)benzylamine. Aust. J. Chem..

[21] Tang Z., Chen W., Zhu Z., Liu H. (2011). Synthesis of 2,3-diaryl-3,4-dihydro-2H-1,3-benzoxazines and their fungicidal activities. J. Heterocyclic Chem..

[22] Xu L.W., Zhou W., Yang L., Xiao C.G. (2007). Chlorotrimethylsilane: A powerful Lewis acidic catalyst in Michael-type Friedel-Crafts reactions of indoles and enones. Synth. Commun..

[23] Xu L.W., Xia C.G. (2004). Highly efficient phosphine-catalyzed aza-Michael reactions of a,b-unsaturated compounds with carbamates in the presence of TMSCl. Tetrahedron Lett..

[24] Xu L.W., Xia C.G., Hu X.X. (2003). An efficient and inexpensive catalyst system for the aza-Michael reactions of enones with carbamates. Chem. Commun..

[25] Tang Z. (2006). Development of silicon-based Lewis acids and their applications to organic synthesis. Chin. J. Org. Chem..

[26] Palmieri G. (1999). Synthesis of enantiopure *o*-hydroxybenzylamines by stereoselective reduction of 2-imidoylphenols: Application in the catalytic enantioselective addition of diethylzinc to aldehydes. Eur. J. Org. Chem..

[27] Cimarelli C., Palmieri G., Volpini E. (2001). Ready N-alkylation of enantiopure aminophenols: Synthesis of tertiary aminophenols. Tetrahedron.

[28] Liu A., Ou X., Huang M., Wang X., Liu X., Wang Y., Chen C., Yao J. (2005). Synthesis and insecticidal activities of novel oxime ether pyrethroids. Pest Manag. Sci..

